# Splenic artery fenestration – a previously undescribed arterial variant

**DOI:** 10.1007/s00276-026-03886-y

**Published:** 2026-04-29

**Authors:** George Triantafyllou, Nikolaos-Achilleas Arkoudis, Ornella Moschovaki-Zeiger, Georgios Velonakis, Dimitrios K. Filippiadis, Maria Piagkou

**Affiliations:** 1 Department of Anatomy, School of Medicine, Faculty of Health Sciences, National and Kapodistrian University of Athens, 75 Mikras Asias str, Goudi, Athens, 11527 Greece; 2 “VARIANTIS” Research Laboratory, Department of Clinical Anatomy, Mazovian Academy in Płock, Płock, Poland; 3 Research Unit of Radiology and Medical Imaging, National and Kapodistrian University of Athens, Goudi, Athens, Greece; 4 Second Department of Radiology, General University Hospital “Attikon”, National and Kapodistrian University of Athens, Goudi, Athens, Greece

**Keywords:** splenic artery, arterial fenestration, anatomical variation, computed tomography angiography

## Abstract

**Purpose:**

The splenic artery exhibits considerable morphological variability; however, true arterial fenestrations are exceedingly rare within the abdominal vasculature. We report a previously undocumented splenic artery fenestration identified on computed tomography angiography (CTA) in a 70-year-old male.

**Anatomic Variation:**

The artery followed an intrapancreatic course and demonstrated a true fenestration located 33.1 mm from its origin. The fenestrated segment consisted of a superior limb measuring 6.1 mm in diameter and an inferior limb measuring 6.1 mm, with distal reconstitution into a single lumen over a length of 15.4 mm. The finding was consistently visualized using multiplanar reconstructions and three-dimensional volume-rendered CTA images, without evidence of mural irregularity, intimal flap, or arterial dissection.

**Conclusions:**

Recognition of this rare arterial configuration is important for radiologists, surgeons, and interventionalists, as it may mimic arterial duplication or dissection and has potential implications for endovascular and surgical procedures involving the splenic artery.

**Supplementary Information:**

The online version contains supplementary material available at 10.1007/s00276-026-03886-y.

## Introduction

Typically, the celiac trunk gives rise to the left gastric artery, common hepatic artery, and splenic artery (SA). The SA courses along the superior border of the pancreas toward the splenic hilum, following a characteristically tortuous path. Along its course, it gives rise to multiple pancreatic and gastric branches, including the dorsal pancreatic artery and the right gastroepiploic artery [1].

Comprehensive anatomic surveys have documented substantial variability of the SA with respect to its origin, course, tortuosity, and branching pattern. Bergman’s *Comprehensive Encyclopedia of Human Anatomic Variations* describes variants arising from alternative arterial trunks, such as hepatosplenic or gastrosplenic configurations, as well as rare origins from the superior mesenteric artery or directly from the abdominal aorta. In addition, marked tortuosity with multiple loops or coils is frequently encountered [2].

Arterial fenestration represents a distinct vascular configuration in which a single artery divides into two parallel lumina that subsequently rejoin, creating a double-lumen segment. This morphology is thought to result from incomplete fusion during vascular development. Fenestrations are most commonly reported in intracranial circulation, particularly within the cerebral arterial circle, where they are well recognized on angiographic studies [3].

In contrast, arterial fenestrations involving the abdominal vasculature are exceptionally rare. Isolated reports have described fenestration of the renal artery and, less frequently, venous fenestration of the renal vein [4, 5]. To our knowledge, true fenestration of the splenic artery has not been previously documented in anatomical or radiologic literature.

## Case report

During a retrospective review of abdominal computed tomography angiography (CTA) examinations, the scan of a 70-year-old male patient demonstrated an unusual SA morphology. The patient had no history of abdominal pathology or prior abdominal surgery. The CTA was performed using a helical high-speed, low-dose scanner (SOMATOM go.Top, 128-slice configuration, Siemens), following the injection of 60 mL of a 30% iodinated contrast solution at a flow rate of 4–4.5 mL/s. Image analysis was performed using Horos software (version 3.3.6; Horos Project). Evaluation included axial source images, multiplanar reconstructions in the coronal and sagittal planes, maximum intensity projections, and three-dimensional volume-rendered reconstructions.

The coeliac trunk was identified as the first anterior branch of the abdominal aorta and measured 7.5 mm in diameter. The left gastric artery originated 12.6 mm distal to the celiac trunk origin. After an additional 4.2 mm, the celiac trunk bifurcated into the common hepatic artery and the SA.

The SA demonstrated an initial diameter of 5.8 mm and followed a tortuous intrapancreatic course before extending laterocranially toward the splenic hilum. At a distance of 33.1 mm from its origin, a true arterial fenestration was identified, forming two distinct lumina that subsequently reconstituted into a single vessel. The superior limb measured 6.1 mm in diameter, while the inferior limb measured 6.1 mm. The fenestrated segment extended over a length of 15.4 mm.

**Fig. 1:**
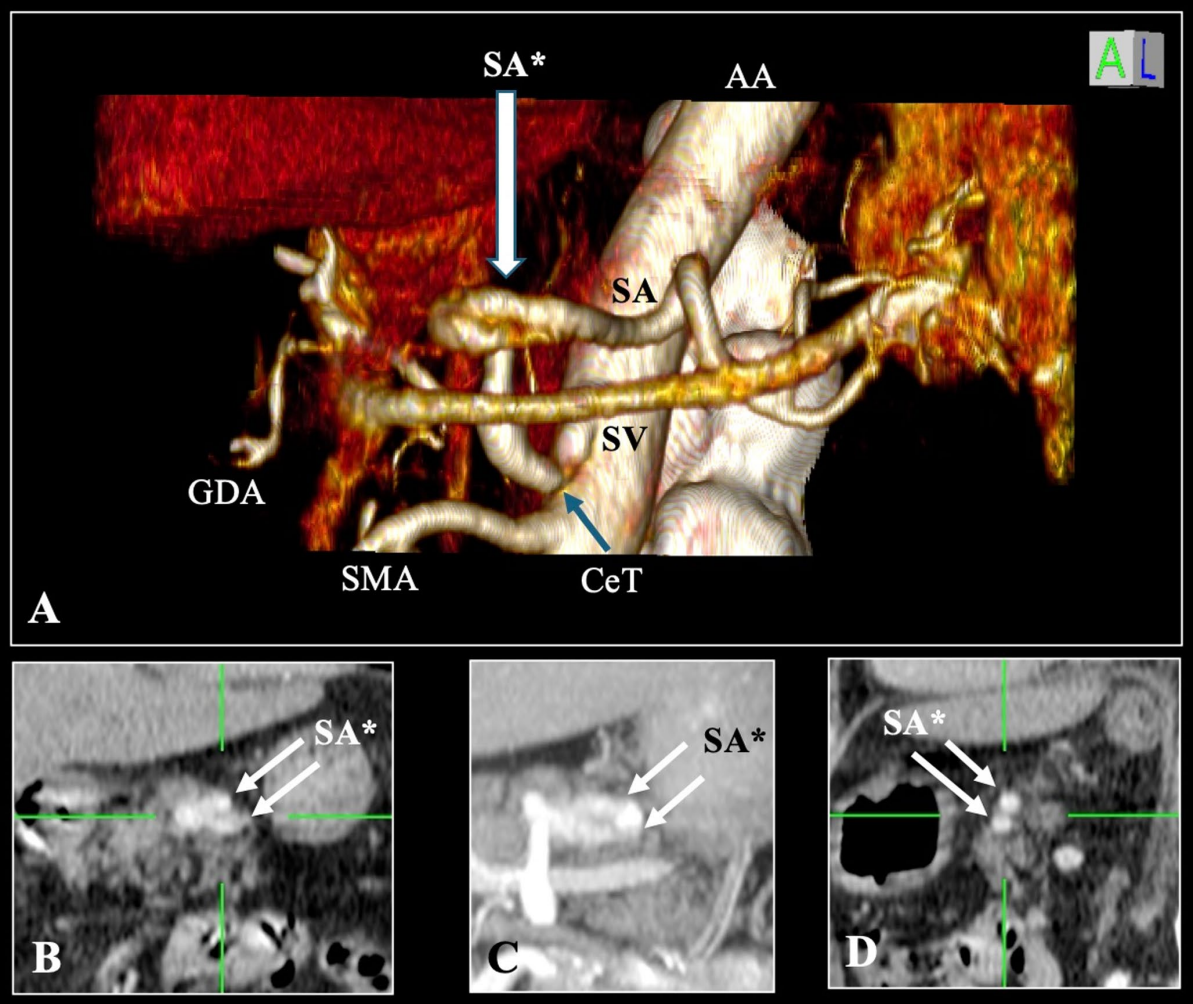
Splenic artery fenestration on computed tomography (CT) angiography. (A) Three-dimensional volume-rendered CT angiography reconstruction demonstrating fenestration of the splenic artery (SA*), with separation into two parallel lumina and distal reconstitution into a single vessel. **(B**,** C)** Coronal multiplanar and maximum intensity projection images confirming the presence of two distinct splenic artery channels (arrows). **(D)** Sagittal multiplanar reconstruction illustrates the longitudinal extent of the fenestrated segment (arrows). AA, abdominal aorta; CT, celiac trunk; SMA, superior mesenteric artery; GDA, gastroduodenal artery; SV, splenic vein

The fenestration was clearly visualized on three-dimensional volume-rendered images (Fig. 1A) and confirmed on coronal (Fig. 1B and C) and sagittal (Fig. 1D) reconstructions. Due to the longitudinal orientation of the fenestrated segment relative to the imaging plane, the finding was not appreciable on axial source images. No mural irregularity, intimal flap, focal stenosis, aneurysmal dilation, or intraluminal thrombus was identified in either limb of the fenestration.

## Discussion

Fenestrations of the vascular system are thoroughly documented within the intracranial circulation and are traditionally associated with the cerebral arterial circle. Indeed, Bergman’s *Comprehensive Encyclopedia of Human Anatomic Variations* reports fenestration predominantly in the context of cerebral arteries [2].

Conversely, fenestrations involving the abdominal vasculature are exceedingly rare, with only sporadic instances documented in the literature. The renal vessels constitute the most frequently reported abdominal sites, including fenestration of the left renal vein, observed in up to 3.34% of cases within a radioanatomical study [5], along with isolated angiographic findings involving the right renal vein [6]. Fenestration of the renal artery has also been documented in a cadaveric study [4]. To date, true fenestration of the SA has not been reported within the radiologic literature.

The present case broadens the spectrum of recognized variations of the SA, thereby emphasizing the significant morphological diversity of this vessel. Variations in the origin, course, and terminal branching patterns are well documented. Although it most frequently originates from the celiac trunk, infrequent origins directly from the abdominal aorta or the superior mesenteric artery have been reported [7]. The SA is characteristically tortuous, with the majority of cases exhibiting at least one loop along its course. Although a suprapancreatic or retropancreatic trajectory is most frequently observed, less common intrapancreatic or anteropancreatic courses have also been documented, analogous to the configuration observed in the current case [8]. At the splenic hilum, the branching patterns exhibit considerable variability, with bifurcation into superior and inferior lobar arteries being the most prevalent, followed by trifurcation and quadrifurcation, with occasional occurrence of an accessory SA [8, 9].

From a radiologic perspective, accurate identification of SA fenestration requires careful differentiation from other entities, including arterial duplication, focal arterial dissection, and overlapping tortuosity. In the present case, duplication was excluded by the presence of a single arterial origin with distal reconstitution into a solitary lumen. Dissection was considered unlikely due to the absence of an intimal flap, mural irregularity, caliber discrepancy, or downstream perfusion abnormality. Consistent visualization of two distinct lumina across multiplanar and three-dimensional reconstructions supports the diagnosis of a true arterial fenestration rather than an acquired or artifactual phenomenon.

The recognition of SA fenestration holds significant clinical importance for both surgeons and interventional radiologists. The course of the artery relative to the pancreas is a critical factor in pancreatic and splenic surgical interventions, particularly when an intrapancreatic trajectory mandates parenchymal dissection for vascular control [8]. From an interventional perspective, fenestrations present unique technical nuances. During catheter-based interventions for splenic trauma or aneurysms, the bifurcation–reunification point can act as a mechanical trap for guidewires, potentially being misinterpreted as a focal dissection or an intimal flap on digital subtraction angiography. Furthermore, if SA embolization is required, the dual channels may necessitate the embolization of both limbs to ensure complete devascularization [10].

## Conclusions

This case identifies a previously undocumented variant of the SA. Our findings demonstrate a unique fenestration that is easily overlooked on standard axial imaging. The application of multiplanar and three-dimensional CTA reconstructions is essential for the accurate diagnosis of this anomaly and its differentiation from pathological or artifactual entities. For the clinician, recognizing this specific fenestration is vital for ensuring complete vascular control and avoiding iatrogenic injury during splenic and pancreatic surgical or endovascular interventions.

## Supplementary Information

Below is the link to the electronic supplementary material.

Supplementary Material 1

## Data Availability

Please contact the authors for data requests.
